# Phosphorylation and Dephosphorylation of Tau Protein During Synthetic Torpor

**DOI:** 10.3389/fnana.2019.00057

**Published:** 2019-06-06

**Authors:** Marco Luppi, Timna Hitrec, Alessia Di Cristoforo, Fabio Squarcio, Agnese Stanzani, Alessandra Occhinegro, Pierfrancesco Chiavetta, Domenico Tupone, Giovanni Zamboni, Roberto Amici, Matteo Cerri

**Affiliations:** ^1^Department of Biomedical and Neuromotor Sciences, University of Bologna, Bologna, Italy; ^2^Department of Veterinary Medical Sciences, University of Bologna, Bologna, Italy; ^3^Department of Neurological Surgery, Oregon Health & Science University, Portland, OR, United States

**Keywords:** rat, hypothermia, hibernation, brain structures, raphe pallidus, tauopathies, adaptive response

## Abstract

Tau protein is of primary importance for many physiological processes in neurons, where it affects the dynamics of the microtubule system. When hyperphosphorylated (PP-Tau), Tau monomers detach from microtubules and tend to aggregate firstly in oligomers, and then in neurofibrillary tangles, as it occurs in a group of neurodegenerative disorders named thauopathies. A hypothermia-related accumulation of PP-Tau, which is quickly reversed after the return to normothermia, has been shown to occur in the brain of hibernators during torpor. Since, recently, in our lab, a hypothermic torpor-like condition (synthetic torpor, ST) was pharmacologically induced in the rat, a non-hibernator, the aim of the present work was to assess whether ST can lead to a reversible PP-Tau accumulation in the rat brain. PP-Tau was immunohistochemically assessed by staining for AT8 (phosphorylated Tau) and Tau-1 (non-phosphorylated Tau) in 19 brain structures, which were chosen mostly due to their involvement in the regulation of autonomic and cognitive functions in relation to behavioral states. During ST, AT8 staining was strongly expressed throughout the brain, while Tau-1 staining was reduced compared to control conditions. During the following recovery period, AT8 staining progressively reduced close to zero after 6 h from ST. However, Tau-1 staining remained low even after 38 h from ST. Thus, overall, these results show that ST induced an accumulation of PP-Tau that was, apparently, only partially reversed to normal during the recovery period. While the accumulation of PP-Tau may only depend on the physicochemical characteristics of the enzymes regulating Tau phosphorylation, the reverse process of dephosphorylation should be actively regulated, also in non-hibernators. In conclusion, in this work a reversible and widespread PP-Tau accumulation has been induced through a procedure that leads a non-hibernator to a degree of reversible hypothermia, which is comparable to that observed in hibernators. Therefore, the physiological mechanism involved in this process can sustain an adaptive neuronal response to extreme conditions, which may however lead to neurodegeneration when particular intensities and durations are exceeded.

## Introduction

Tau protein is a microtubule-associated protein that is predominantly present in neurons, where it is of primary importance for many physiological processes due to its effects on the dynamics of the microtubule system (Wang and Mandelkow, 2016).

Tau tends to dissociate from microtubules when a hyperphosphorylation (PP-Tau) substitutes the bound physiological configuration of two phosphates per protein molecule (Wang and Mandelkow, 2016). In certain conditions and hyperphosphorylation states that are still largely unknown, monomers of Tau aggregate in oligomers that, either by coalescing in neurofibrillary tangles (NFT) or spreading as neurotoxic agents, characterize a group of disorders defined as tauopathies, ranging from Alzheimer’s disease (AD) to neurodegenerative pathologies (Gerson et al., 2016; Kovacs, 2017).

However, PP-Tau has been evidenced not only in tauopathy subjects, but also in hamsters during torpor (Arendt et al., 2003), as well as in mice (facultative hibernators; Hudson and Scott, 1979) exposed to different physiological challenges, such as starvation (Yanagisawa et al., 1999; Planel et al., 2001), cold water stress (Okawa et al., 2003), or general anesthesia (Planel et al., 2007). Remarkably, in all these conditions, PP-Tau accumulation has been shown to be reversible.

The hypothermia that follows a decline in metabolic rate appears to be key for PP-Tau induction (Arendt et al., 2015). This was confirmed in mice made hypothermic either by starvation, or by the i.p. administration of insulin or deoxyglucose (Planel et al., 2004). Also, PP-Tau induction was suppressed in mice that were kept euthermic while under general anesthesia (Xiao et al., 2013).

The dynamics of hypothermia-related phosphorylation/dephosphorylation of Tau protein, which have been widely studied in either obligate or facultative hibernators, still have to be clearly assessed in non-hibernating species. In fact, only an indirect assessment of the degree of Tau phosphorylation has been carried out in rats, suggesting a two–threefold increase in several brain areas following cold-water stress exposure (Korneyev et al., 1995). Recently, in our lab (Cerri et al., 2013), a deep and fully reversible hypothermic state was induced in the rat by the direct inhibition of the raphe pallidus (RPa), the brain region containing the premotor sympathetic neurons that control thermogenesis (Morrison and Nakamura, 2018). This condition, defined as “synthetic torpor” (ST; Cerri, 2017), is characterized by a decrease in body temperature that, being induced by the central blockade of the final common pathway of thermogenesis, is physiologically more similar to the process that occurs in natural torpor-hibernation than when it is induced by an external body cooling procedure that overwhelms thermoregulatory responses.

The aim of the present work was to assess whether the induction of ST in the rat coexisted with a reversible accumulation of PP-Tau throughout the brain. To this end, Tau phosphorylation was immunohistochemically assessed in ST during the induction phase and the following recovery in neural structures related to the regulation of autonomic (15) and cognitive (4) functions in behavioral states.

## Materials and Methods

### Animals

A total of 19 Male Sprague–Dawley rats (201–225 g; Charles River) were used. Animals were acclimated to normal laboratory conditions: ambient temperature (Ta) set at 24 ± 0.5°C; 12 h:12 h light-dark (LD) cycle (L: 09:00 h–21:00 h; 100–150 lux at cage level); food and water *ad libitum*. All the experiments were conducted following the approval by the National Health Authority (decree: No. 112/2018-PR), in accordance with the DL 26/2014 and the European Union Directive 2010/63/EU, and under the supervision of the Central Veterinary Service of the University of Bologna. All efforts were made to minimize the number of animals used and their pain and distress.

### Surgery

The procedure has been previously described (Cerri et al., 2013). Briefly, deeply anesthetized rats (Diazepam, 5 mg/kg i.m.; Ketamine-HCl, 100 mg/kg i.p.) were placed in a stereotaxic apparatus (David Kopf Instruments) and surgically implanted with: (i) electrodes for the electroencephalogram (EEG); (ii) a thermistor (Thermometrics Corporation) mounted inside a stainless steel needle (21 gauge) and placed beside the left anterior hypothalamus to record the deep brain temperature (Tb); (iii) a microinjection guide cannula, targeted to the RPa, at the following coordinates from lambda: on the midline, 3.0 mm posterior and 9.0 ventral to the dorsal surface of the cerebellum (Paxinos and Watson, 2007). After surgery, animals received 20 ml/kg saline subcutaneously and 0.25 ml of an antibiotic solution (penicillin G and streptomycin-sulfate) intramuscularly. Each rat recovered from surgery for at least 1 week. Prior to the experimental session rats were placed in a cage positioned within a thermoregulated and sound-attenuated chamber. This adaptation period was 3 days long, during which rats were exposed to a mild low Ta (15°C), constant darkness and were fed a high-fat diet (35% fats, Mucedola), conditions that are known to favor the occurrence of a torpid state in hibernators (Cerri et al., 2013).

### Synthetic Torpor

To induce ST, we used the consolidated protocol proposed by Cerri et al. (2013). Briefly, a microinjecting cannula was inserted into the guide cannula placed just above the RPa. Then, 100 nl of the GABA_A_ agonist muscimol (1 mM) was injected once an hour, six consecutive times. Following the last injection, Tb reached values of around 22°C (Cerri et al., 2013). At 17.00 h, 1 h after the last injection, Ta was set at 28 ± 0.5°C to favor the return to normothermia of the animal. A group of animals (Controls) were injected with artificial cerebrospinal fluid (aCSF; EcoCyte Bioscience). During the whole experiment, EEG and Tb signals were recorded, after being opportunely amplified, filtered, and digitalized (Cerri et al., 2013), at the aim of better monitoring animals’ behavior during ST induction and in the following recovery period.

### Experimental Procedure

**FIGURE 1:**
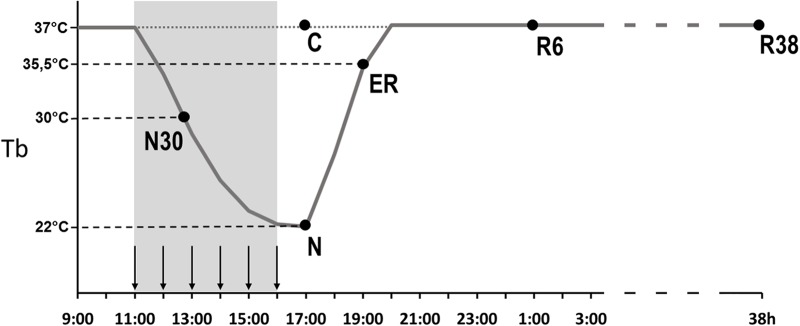
Schematic representation of the experimental procedure. The solid line indicates the progress of brain temperature (Tb) throughout the experiment. The dotted line refers to the control group (C). The gray area represents the time period during which the synthetic torpor (ST) was induced, arrows indicate the injections (see section “Materials and Methods”). N30, samples taken when Tb reached 30°C; N, samples taken at nadir of hypothermia, during the ST; ER, early recovery, samples taken when Tb reached 35.5°C following ST; R6, samples taken 6 h after ER; R38, samples taken 38 h after ER.

Animals were randomly assigned to six different experimental groups and were sacrificed at different times following the injection of either muscimol or aCSF (first injection at 11.00 h). Tb levels at the moment of the sacrifice are shown in Figure 1, for each group. The experimental groups were the following:

- C → Control, injected with aCSF (*N* = 2) and sacrificed at around 17.00 h, exactly matching the N condition.
- N30 → sacrificed at around 12.00 h–13.00 h, between the second and third injection of muscimol, when Tb reached the level of 30°C (*N* = 3).
- N → sacrificed 1 h after the last injection, at 17.00 h, when Tb reached the nadir of hypothermia (*N* = 3; Tb = 22.1 ± 1.4°C).
- ER → early recovery; sacrificed at around 19.00 h (2 h after Ta was moved from 15 to 28°C) when Tb reached 35.5°C after ST; at this specific point of the protocol, animals began to show clear signs of sleep at the EEG level (*N* = 4).
- R6 → sacrificed at around 01.00 h, 6 h after ER (*N* = 4).
- R38 → sacrificed at around 09.00 h of the third day, 38 h after ER (*N* = 3).

### Immunohistochemistry

At the different time points of the experimental protocol, rats under general anesthesia were transcardially perfused with 200 ml of saline solution (NaCl 0.9%, w/v) followed by an equal amount of 4% (w/v) paraformaldehyde solution in sodium phosphate buffer (PBS). The brain and spinal cord were extracted and post-fixed for 2 h by immersion in the same solution used for the perfusion. Then, both samples were put overnight in a 30% (w/v) sucrose solution in PBS and sodium-azide 0.02% (w/v) for cryoprotection. Hereafter, tissue samples were embedded in a cryostat cutting medium (Killik) and cut into 35 μm-thick slices using a cryostat-microtome (Frigocut 2800), kept at -22.0°C. All the slices were then stored, until analyzed, at -80°C in a cryoprotectant solution: 30% (w/v) sucrose, 30% (v/v) ethylene glycol, 1% (w/v) polyvinylpyrrolidone in PBS.

A sample of one out of every six slices of the whole brain was used for immunostaining. Slices were rinsed twice in PBS and then incubated for 2 h in 1% (v/v) normal donkey serum. Consequently, all slices were incubated overnight with the following primary antibodies: (i) monoclonal rabbit Anti-NeuN (Merck-Millipore), a neuronal marker; (ii) monoclonal mouse Anti-AT8 (Thermo Fisher), marker of the phospho-[Ser202/Thr205]-Tau protein. Both primary antibodies were diluted at 1:400. Slices were then rinsed twice in PBS with 0.3% (v/v) Triton X-100 and incubated with the following secondary antibodies: (i) Donkey Anti-rabbit IgG conjugated with Alexa-488 (Thermo Fisher); (ii) Donkey Anti-mouse IgG conjugated with Alexa-594 (Thermo Fisher). Both secondary antibodies were diluted at 1:500. Finally, tissue slices were mounted on coated glass slices and coverslipped with an anti-fade mounting medium (ProLong Gold mountant; Thermo Fisher).

As a control for the AT8 detection, the same procedure was carried out using the monoclonal mouse Anti-Tau-1 (Merck-Millipore; 1:400), followed by a Donkey Anti-mouse IgG conjugated with Alexa-594 (1:500), that detects Tau protein when it has no phosphorylation between residues from 189 to 207 (Szendrei et al., 1993; Billingsley and Kincaid, 1997). For this procedure, only samples from C, N, and R38 were analyzed.

Since PP-Tau appears to be associated with neuroinflammation (Davies et al., 2017; Nilson et al., 2017), we also decided to assess the degree of activation of parenchymal microglia in a small subgroup of experimental slide sets. The activation state of microglia was evaluated qualitatively by analyzing their morphology (Orr et al., 2009), following the specific staining with the rabbit polyclonal Anti-Iba1 antibody (1:800; Wako) and the secondary antibody Anti-rabbit IgG conjugated with Alexa-488. The procedure was the same as that described earlier in the text. The analysis of microglia activation was conducted in one animal from each of the following experimental conditions: C, N, R6, and R38. Only the following structures were analyzed: paraventricular nucleus of the hypothalamus, CA3 field of the hippocampus, and parietal cortex.

### Image Acquisition and Analysis

**FIGURE 2:**
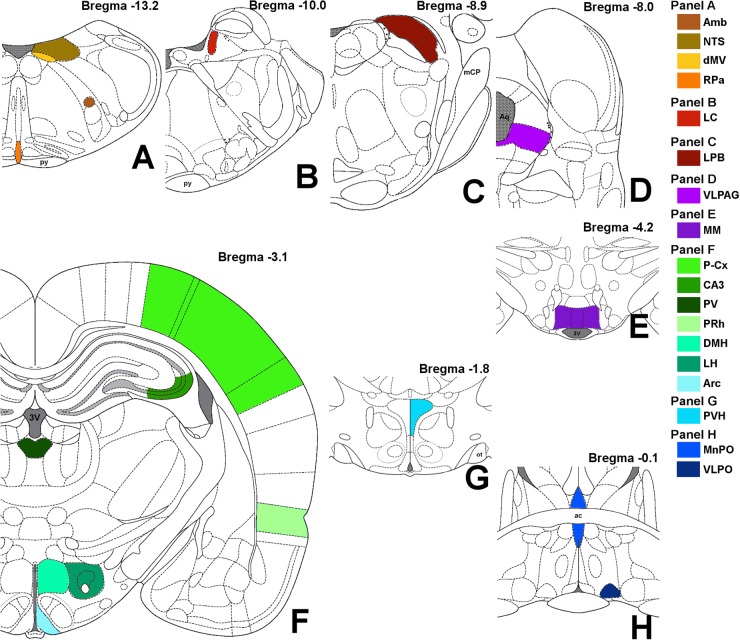
Schematic representation of the brain areas analyzed. Bregma level is reported on each panel; see also Table 1 for more details. Panel **A**: Amb, nucleus ambiguus; NTS, nucleus of the solitary tract; dMV, dorsal motor nucleus of the vagus nerve; RPa, raphe pallidus; py, pyramidal tract. Panel **B**: LC, locus coeruleus. Panel **C**: LPB, lateral parabrachial nucleus; mCP, middle cerebellar peduncle. Panel **D**: VLPAG, ventrolateral part of the periaqueductal gray matter; Aq, Sylvius aqueduct. Panel **E**: MM, medial mammillary nuclei; 3V, third ventricle. Panel **F**: LH, lateral hypothalamus; Arc, arcuate nucleus of the hypothalamus; DMH, dorsomedial nucleus of the hypothalamus; PV, paraventricular nucleus of the thalamus; CA3, field CA3 of the hippocampus; PRh, perirhinal cortex; P-Cx, parietal cortex. Panel **G**: PVH, paraventricular nucleus of the hypothalamus; ot, optic tract. Panel **H**: VLPO, ventrolateral preoptic nucleus; MnPO, median preoptic nucleus of the hypothalamus; ac, anterior commissure.

Images were obtained with a Nikon eclipse 80i equipped with Nikon Digital Sight DS-Vi1 color camera, at 100× magnification (200× for the microglia staining). The 19 brain areas analyzed are shown in Figure 2, as a diagram of their anatomical location and listed in Table 1 in a caudal to rostral direction: nucleus ambiguus (Amb); dorsal motor nucleus of the vagus nerve (dMV); nucleus of the solitary tract (NTS); raphe pallidus (RPa); locus coeruleus (LC); lateral parabrachial nucleus (LPB); ventrolateral part of the periaqueductal grey matter (VLPAG); medial mammillary nucleus (MM); lateral hypothalamus (LH); arcuate nucleus of the hypothalamus (Arc); dorsomedial nucleus of the hypothalamus (DMH); paraventricular nucleus of the hypothalamus (PVH); ventrolateral preoptic nucleus (VLPO); median preoptic nucleus of the hypothalamus (MnPO); paraventricular nucleus of the thalamus (PV); cerebellum cortex (Cb-Cx); CA3 field of the hippocampus (CA3); perirhinal cortex (PRh); parietal cortex (P-Cx). The visual recognition of these structures was possible while observing NeuN staining (Alexa-488) of the whole section and comparing it with the atlas schemes. In particular, the experimenter at the microscope was able to define the exact field to be digitally acquired by easily recognizing some clear and unmistakable neuroanatomical structures, such as: (i) for the brainstem: the shape of the forth ventricle, the pyramidal tracts, the complex of cochlear nuclei, the inferior olive complex of nuclei, the facial nucleus, the typical caudo-rostral developing shape of the Sylvius aqueduct, the typical shape of the middle cerebellar peduncle; (ii) for the diencephalic structures: the shape of the third ventricle, the lateral and dorso-ventral extension of the hippocampus, the position of the fornix, the optic chiasm or the shape of the optic tracts, the anterior commissure.

**Table 1:** Brain structures analyzed.

Brain structure		Abbreviation	Bregma level^∗^	Abbreviation(s) as in Paxinos and Watson (2007)^∗^	Functional involvement	Suggested references	Panel in Figure 2
Medulla	Nucleus ambiguus	Amb	-13/-13,5	AmbSC	Autonomic function – Parasympathetic	Brailoiu et al., 2017	A
	Dorsal motor nucleus of the vagus nerve	dMV	-13/-13,5	10N	Autonomic function – Parasympathetic	Hopkins et al., 1996	A
	Nucleus of the solitary tract	NTS	-13/-13,5	SolI; sol; SolC; SolIM; SolCe; SolL; SolDL; SolM; SolV; SolVL; Psol	Visceral sensory integration – Central autonomic regulation	Zoccal et al., 2014	A
	**Raphe pallidus**	**Rpa**	-13/-13,5	Rpa	**Thermoregulation**	Morrison and Nakamura, 2018	A
Pons	Locus coeruleus	LC	-9,6/-10,1	LC	Behavioral state control	Benarroch, 2018	B
	**Lateral parabrachial nucleus**	**LPB**	-8,9/-9,2	LPBD; LPBCr; LPBE; LPBV; LPBI; LPBC	Central autonomic regulation – **Thermoregulation**	Morrison and Nakamura, 2018	C
Midbrain	Ventrolateral part of the periaqueductal gray matter	VLPAG	-8,0/-8,5	VLPAG	Central autonomic regulation – Behavioral state control	Luppi, 2010	D
Hypothalamus	Medial mammillary nucleus	MM	-4,2/-4,4	MnM; MM	Memory formation and consolidation	Vann and Nelson, 2015	E
	Lateral hypothalamus	LH	-3,0/-3,4	PeFLH; PeF	Behavioral state control – Regulation of body metabolism	Luppi, 2018	F
	Arcuate nucleus	Arc	-3,0/-3,4	ArcD; ArcM; ArcL	Regulation of body metabolism	Wu et al., 2014	F
	**Dorsomedial nucleus**	**DMH**	-3,0/-3,4	DMC; DMD; DMV	**Thermoregulation**	Morrison and Nakamura, 2018	F
	**Paraventricular nucleus**	**PVH**	-1,6/-1,9	PaMM; PaMP; PaV; PaDC; PaLM	Central autonomic regulation – Osmoregulation-**Thermoregulation**	McKinley et al., 2015; Morrison and Nakamura, 2018	G
	**Ventrolateral preoptic nucleus**	**VLPO**	-0,0/-0,6	VLPO	Behavioral State Control – **Thermoregulation**	Luppi, 2010	H
	**Median preoptic nucleus of the hypothalamus**	**MnPO**	-0,1/-0,2	MnPO	Central autonomic regulation – Behavioral State Control – Osmoregulation-**Thermoregulation**	McKinley et al., 2015; Morrison and Nakamura, 2018	H
Thalamus	Paraventricular nucleus	PV	-3,0/-3,4	PVP	Central autonomic regulation	Colavito et al., 2015	F
Cerebellum	Cerebellar cortex	Cb-Cx	-11,5/-13,5	General sample picture from cortical layers	Motor functions –Learning – Lack of Tau phosphorylation in tauopathies	Hu et al., 2017	n.s.
Hippocampus	CA3 field	CA3	-3,0/-3,4	CA3; SLu; Rad	Memory formation and consolidation – Well studied for assessing Tau phosphorylation in hibernators	Arendt et al., 2003, 2015	F
Brain Cortex	Perirhinal cortex	PRh	-3,0/-3,4	PRh	Memory formation and consolidation – Recognition of environmental stimuli	Suzuki, 1996	F
	Parietal cortex	P-Cx	-3,0/-3,4	Broadly covering primary somatosensory cortex (S1Tr; S1DZ; S1BF; S1ULp)	Sample of neocortex – Sensory integration – Well studied for assessing Tau phosphorylation in hibernators	Arendt et al., 2003, 2015	F


*List of the brain neural structures analyzed, arranged in a caudal to rostral order. Structures directly involved in thermoregulation are shown in bold.*

Following recognition, each microscopic field of interest was acquired in two separate pictures that were distinct according to the fluorochrome used, in order to have NeuN (Alexa-488) for the anatomical identification and AT8 or Tau-1 (Alexa-594) for the PP-Tau staining for each picture taken. In order to create digital pictures with the best quality reproduction of the variations of staining intensity observed for AT8 and Tau-1 at the microscope, the exposure time of the camera was manually regulated for each picture to the best of the experimenter evaluations; the aim was to best reproduce on the taken picture what observed directly through the oculars. The experimenter was blind to the experimental conditions. The fine regulation of the exposure time for every picture was necessary to avoid automatic compensations of the camera, due to the dark field of fluorescence images. All the other camera parameters, such as white balance and gain (Y100-R100-B100), were kept constant throughout the experiment. Thanks to the preview function of the camera, all this procedure took very few seconds for each picture, avoiding any problem of fluorescence fading.

Analyses were carried out independently by two experimenters working in the same conditions: each experimenter was unaware of the experimental conditions and always used the same digital setup, with fixed brightness and contrast regulations. PP-Tau accumulation was quantified in each AT8 and Tau-1 picture, estimating the staining intensity while observing pictures on the monitor (using Windows Photo Viewer) by giving a score ranging from “-” (completely absent) to “++++” (maximum staining) on a scale of five levels (Supplementary Figure S1). The final score was obtained by averaging the scores given by the two experimenters, considering all the animals belonging to the same experimental condition.

### Statistical Analysis

The analysis consisted in two steps: (i) “gross analysis” considering together the scores of all the structures analyzed; (ii) “fine analysis” considering structures separately.

We used the non-parametric Kruskal-Wallis test and, only if the null hypothesis was rejected, pairwise comparisons were carried out using the non-parametric Mann-Whitney test in the following evaluations: (i) for AT8 staining: all the experimental conditions vs. C; R6 vs. ER; R6 vs. R38; (ii) for Tau-1 staining: all the experimental conditions vs. C; N vs. R38. Significance level was preset at *P* < 0.05 for all comparisons.

No statistical analysis was conducted on the morphological evaluation of the microglia activation.

## Results

### Tau Phosphorylation: Gross Analysis

In this first step of the analysis, for each experimental condition the scores relative to the 19 neural structures were considered together as a whole.

**FIGURE 3:**
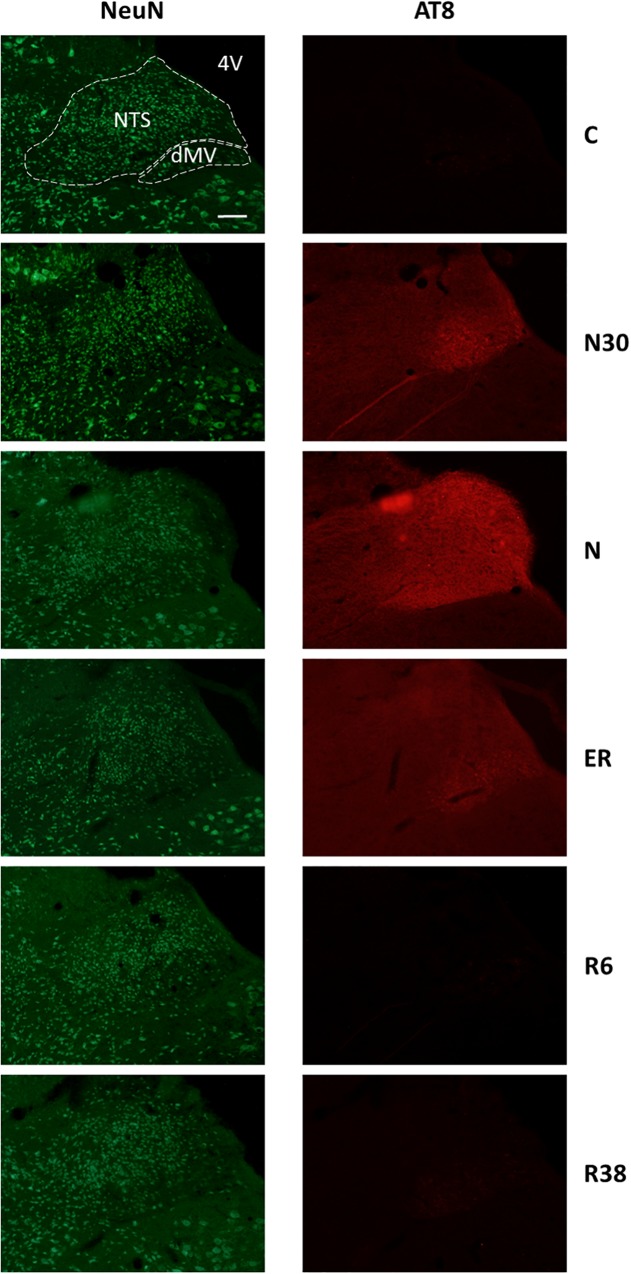
Representative pictures showing the nucleus of the solitary tract (NTS) and the motor nucleus of the vagus (dMV). Left column represents NeuN staining (neuronal maker, secondary conjugated with Alexa-488), for the recognition of the anatomical structures. Only for the top image, the anatomical structures are marked with white dotted lines (calibration bar: 100 μm). Right column represents the same corresponding field depicted in the left column, but stained for AT8 (phosphorylated Tau, secondary conjugated with Alexa-594). C, control; N30, samples taken during the induction of synthetic torpor (ST), when brain temperature (Tb) reached 30°C; N, samples taken at nadir of hypothermia, during the ST; ER, early recovery, samples taken when Tb reached 35.5°C following ST; R6, samples taken 6 h after ER; R38, samples taken 38 h after ER. 4V, fourth ventricle.

**FIGURE 4:**
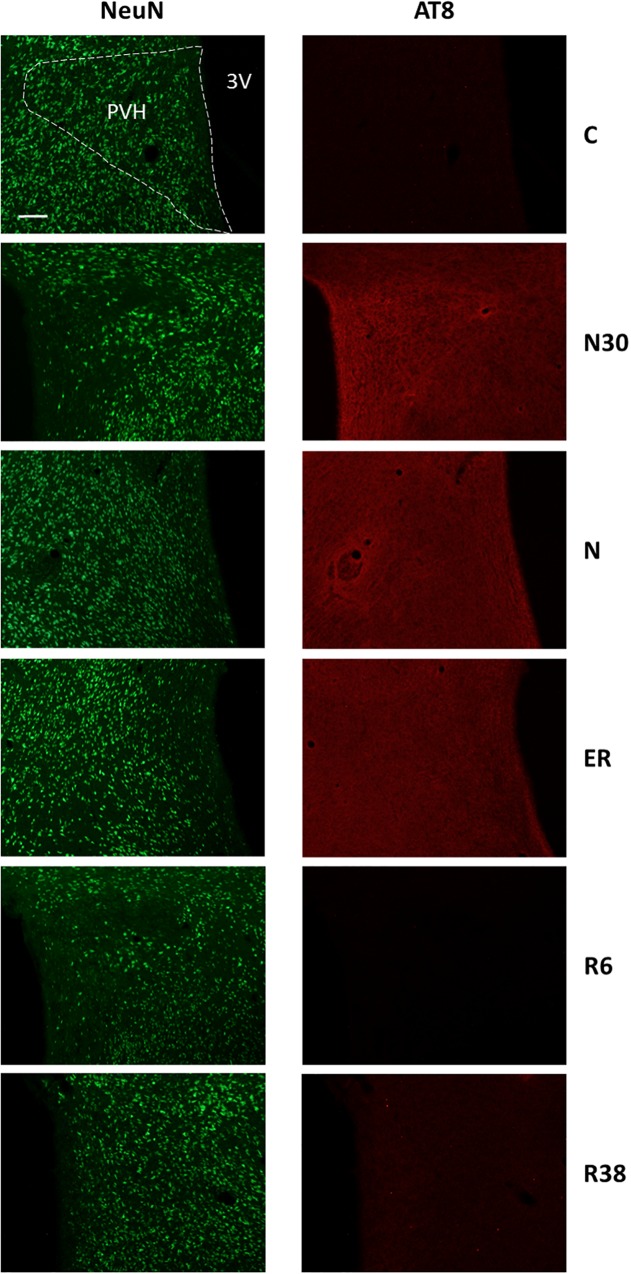
Representative pictures showing the paraventricular nucleus of the hypothalamus (PVH). Left column represents NeuN staining (neuronal maker, secondary conjugated with Alexa-488), for the recognition of the anatomical structure. Only for the top image, the anatomical structure is marked with a white dotted line (calibration bar: 100 μm). Right column represents the same corresponding field depicted in the left column, but stained for AT8 (phosphorylated Tau, secondary conjugated with Alexa-594). C, control; N30, samples taken during the induction of synthetic torpor (ST), when brain temperature (Tb) reached 30°C; N, samples taken at nadir of hypothermia, during the ST; ER, early recovery, samples taken when Tb reached 35.5°C following ST; R6, samples taken 6 h after ER; R38, samples taken 38 h after ER. 3V, third ventricle.

**FIGURE 5:**
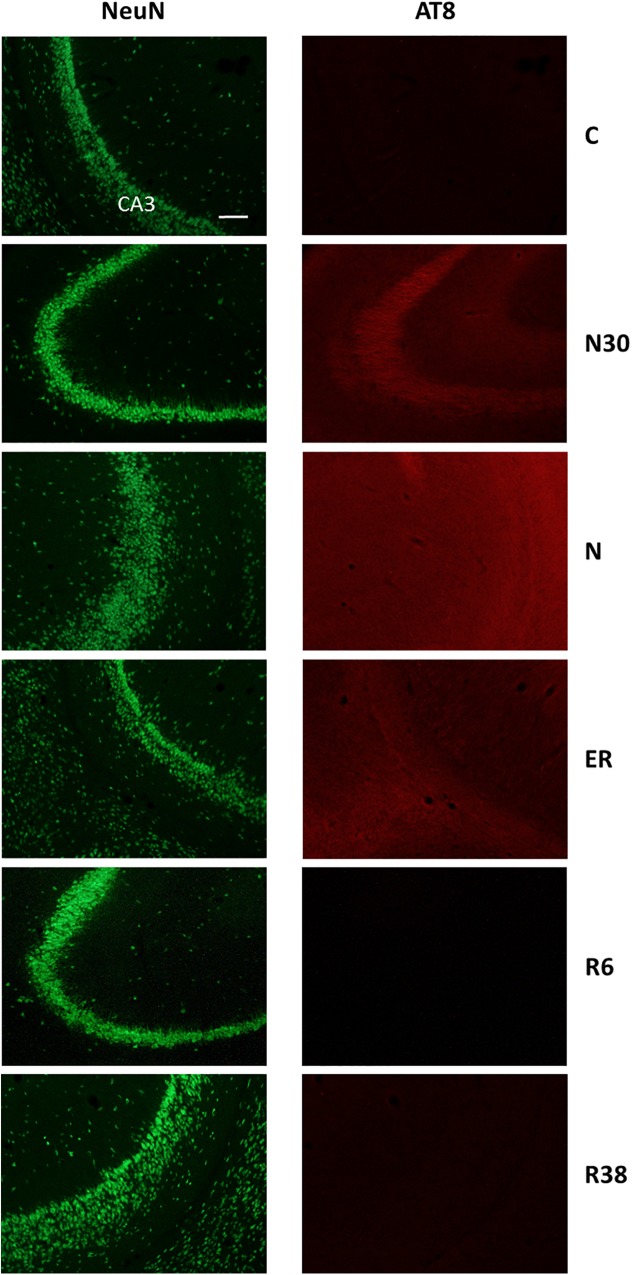
Representative pictures showing the field CA3 of the hippocampus (CA3). Left column represents NeuN staining (neuronal maker, secondary conjugated with Alexa-488), for the recognition of the anatomical structure. Only for the top image, the anatomical structure is identified (calibration bar: 100 μm). Right column represents the same corresponding field depicted in the left column, but stained for AT8 (phosphorylated Tau, secondary conjugated with Alexa-594). C, control; N30, samples taken during the induction of synthetic torpor (ST), when brain temperature (Tb) reached 30°C; N, samples taken at nadir of hypothermia, during the ST; ER, early recovery, samples taken when Tb reached 35.5°C following ST; R6, samples taken 6 h after ER; R38, samples taken 38 h after ER.

When comparing scores obtained for the AT8 staining intensity, as described in Table 2 and shown in the exemplificative Figure 3–5, the results showed that staining levels in N30, N, and ER were significantly higher than in Controls (*P* < 0.001 for each comparison). Also, ER presented higher scores than those found in R6, and this difference was statistically significant (*P* < 0.001). R6 and R38 did not differ from each other (N.S.); nor were they different from C (N.S. for both comparisons).

**Table 2:** AT8 and Tau-1 staining intensities.

	AT8						Tau-1		
		
	C	N30^∗^	N^∗^	ER^∗^	R6^#^	R38	C	N^∗^	R38^∗^
Amb	-	+++	++^∗^	+^∗^	-^#^	-	++	++	+
NTS	+	+	++^∗^	++	-^∗#^	-	++	++	++
dMV	+	++	++^∗^	++	-	-	++	++	++
Rpa	++	++	+++	++	+^#^	++	+	+	-^∗^
LC	-	++	+^∗^	+	-	-	++	++	++
LPB	-	+^∗^	++^∗^	++^∗^	-^#^	-	++	+	+
VLPAG	+	+	+	+	-^∗#^	-	+++	++	++
Cb-Cx	-	+	+^∗^	+	-	-	+	+	+
MM	-	++^∗^	++++^∗^	++^∗^	+^∗#^	+	++	++	++
LH	+	++	+++^∗^	++^∗^	-^∗#^	-	+++	+^∗^	++^∗^
Arc	-	++^∗^	+++^∗^	++^∗^	+^#^	+	+++	++^∗^	++
DM	-	+	+^∗^	+^∗^	-^#^	-	+++	+	++
PVH	-	+++^∗^	+++^∗^	++^∗^	-^#§^	+^∗^	+++	+^∗^	++
PV	-	+	+^∗^	+	-^#^	-	+++	++	++
VLPO	-	+++^∗^	++++^∗^	+^∗^	-^#^	+	+++	++^∗^	++
MnPO	-	++^∗^	+++^∗^	+^∗^	-^#^	-	+++	+^∗^	++
CA3	+	+	+++^∗^	++^∗^	-^∗#^	-	++	+	+
PRh	-	++	+^∗^	+	-^#^	-	++++	++	++^∗^
P-Cx	+	++	+	+	-^∗#^	-	+++	+^∗^	++^∗^


*Brain structures are listed in order from caudal to rostral. See Table 1 and Figure 2 for acronym definition. Staining intensities are represented in a qualitative scale (see Supplementary Figure S1), ranging from “-” (no staining) to “++++” (maximum staining observed). Results are the average of two independent blind evaluations by different experimenters. C, control; N30, samples taken during the induction of synthetic torpor (ST), when brain temperature (Tb) reached 30°C; N, samples taken at nadir of hypothermia, during the ST; ER, early recovery, samples taken when Tb reached 35.5°C following ST; R6, samples taken 6 h after ER; R38, samples taken 38 h after ER. Statistically significant differences (*P* < 0.05) are shown: ^∗^ vs. C; ^#^R6 vs. ER; ^§^ R6 vs. R38.*

**FIGURE 6:**
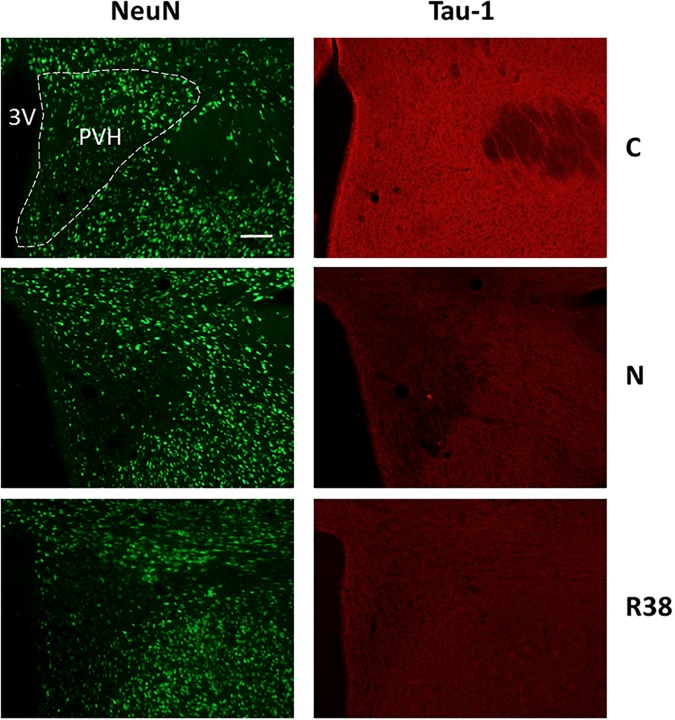
Representative pictures showing the paraventricular nucleus of the hypothalamus (PVH). Left column represents NeuN staining (neuronal maker, secondary conjugated with Alexa-488), for the recognition of the anatomical structure. Only for the top image, the anatomical structure is marked with a white dotted line (calibration bar: 100 μm). Right column represents the same corresponding field depicted in the left column, but stained for Tau-1 (non-phosphorylated Tau, secondary conjugated with Alexa-594). C, control; N, samples taken at nadir of hypothermia, during the synthetic torpor (ST); R38, samples taken 38 h after 35.5°C brain temperature was reached. 3V, third ventricle.

Concerning Tau-1 staining intensity, which is shown in Table 2 and in Figure 6 as an example, the differences were almost the reverse compared to those found for AT8 for both C and N, but not for R38. In particular, staining levels in both N and R38 were significantly lower than those in C (*P* < 0.001 for each comparison). No substantial differences were observed between N and R38.

### Tau Phosphorylation: Fine Analysis

Considering AT8 staining, large and significant variations were observed in the majority of the brain structures analyzed, although the results were rather heterogeneous in the different structures. As far as Tau-1 staining is concerned, only a few neural structures presented statistically significant differences.

For clarity, these results will be described separately for each experimental condition, although the whole scenario is shown in Table 2 for both AT8 and Tau-1.

#### N30 (Samples Taken During the Induction of ST; Figure 1)

In this experimental condition, statistically significant higher AT8 staining levels compared to C were only found in six structures mainly involved in thermoregulatory and metabolic control (Table 1): LPB (*P* = 0.006), MM (*P* = 0.006), Arc (*P* = 0.021), PVH (*P* = 0.008), VLPO (*P* = 0.006), and MnPO (*P* = 0.046).

#### N (Samples Taken During ST, at the Nadir of Tb; Figure 1)

As shown in Table 2, in this experimental condition the large majority of the structures that were analyzed presented a significantly higher staining intensity for AT8 compared to C. In particular: Amb (*P* = 0.003), NTS (*P* = 0.008), dMV (*P* = 0.048), LC (*P* = 0.021), LPB (*P* = 0.006), Cb-Cx (*P* = 0.003), MM (*P* = 0.006), LH (*P* = 0.026), Arc (*P* = 0.007), DMH (*P* = 0.006), PVH (*P* = 0.006), PV (*P* = 0.006), VLPO (*P* = 0.006), MnPO (*P* = 0.008), CA3 (*P* = 0.009), and PRh (*P* = 0.046). In contrast, only a few structures did not show differences compared to C: RPa, VLPAG and P-Cx (NS, for all these comparisons).

Table 2 also shows that the staining intensity of Tau-1 was lower than that found in C for the following structures: LH (*P* = 0.041), Arc (*P* = 0.038), PVH (*P* = 0.046), VLPO (*P* = 0.046), MnPO (*P* = 0.016), and P-Cx (*P* = 0.014).

#### ER (Samples Taken During the Early Recovery From ST, When Tb Reached 35.5°C; Figure 1)

In this condition, the staining intensity found for AT8 was still significantly higher than in C for the majority of the structures in which an hyperphosphorylation was observed in N: Amb (*P* = 0.041), LPB (*P* = 0.005), MM (*P* = 0.005), LH (*P* = 0.026), Arc (*P* = 0.006), DMH (*P* = 0.028), PVH (*P* = 0.003), VLPO (*P* = 0.011), MnPO (*P* = 0.023), and CA3 (*P* = 0.026).

#### R6 (Samples Taken 6 h After ER; Figure 1)

After 6 h of the recovery from the re-attainment of euthermia following ST, significant differences of staining intensity for AT8 compared to C were very limited: in NTS the intensity was significantly lower (*P* = 0.041), the same being true for VLPAG (*P* = 0.041), LH (*P* = 0.041), CA3 (*P* = 0.041), and P-Cx (*P* = 0.041). Only in MM results show a significantly higher (*P* = 0.041) staining intensity compared to C.

Moreover, in order to better assess the dynamics of de-phosphorylation, AT8 staining intensity data from this experimental condition were also compared with ER. The staining intensity resulted significantly lower in R6 in the large majority of the structures analyzed: Amb (*P* = 0.011), NTS (*P* = 0.001), RPa (*P* = 0.020), LPB (*P* < 0.001), VLPAG (*P* = 0.027), MM (*P* = 0.010), LH (*P* < 0.001), Arc (*P* = 0.006), DMH (*P* = 0.030), PVH (*P* < 0.001), PV (*P* = 0.025), VLPO (*P* = 0.009), MnPO (*P* = 0.016), CA3 (*P* < 0.001), PRh (*P* = 0.025), and P-Cx (*P* = 0.003).

#### R38 (Samples Taken 38 h After ER; Figure 1)

Results from this late-recovery condition, regarding the AT8 staining intensity, were very similar to those of C. The only statistically significant difference was that relative to the PVH (*P* = 0.046), in which the staining resulted slightly higher in R38 than in C.

Lastly, AT8 data from this experimental condition were also compared with the R6 condition. Once again the only statistically significant difference was relative to the PVH (*P* = 0.008), with a staining intensity slightly higher in R38 than in R6.

Concerning Tau-1, the staining intensity found in this experimental condition was significantly lower than that found in C for the following structures: RPa (*P* = 0.005), LH (*P* = 0.007), PRh (*P* = 0.008), and P-Cx (*P* = 0.027).

## Discussion

The results of this work show that ST induced a reversible accumulation of PP-Tau in the brain, in a species, the rat, that is unable to spontaneously enter a torpor-hibernation state.

In selected brain areas of rats exposed to cold-water stress, Korneyev et al. (1995) observed a two-fold increase in the immunoreactivity of the Tau paired helical filaments, which normalized 3 h after the end of the stress procedure. In this case, however, both the method of inducing hypothermia and its estimated degree (Hannon, 1958) were far different from those used in our study or from the conditions that occur physiologically in hibernators. Hence, our results may expand the view of hypothermia as a model for studying either the physiology or the pathology of Tau (Wang and Mandelkow, 2016). In fact, the possibility to fully control ST (Cerri et al., 2013; Tupone et al., 2013) permits to handle and to reproduce degrees and durations of hypothermia in non-hibernating mammals that are commonly used in laboratory studies to an extent that is comparable to that of hibernators.

Since we suppose that the development and the resolution of the processes underlying PP-Tau accumulation are driven by different mechanisms, we will discuss them separately.

### Hypothermia-Induced Phosphorylation of Tau Protein

Our results show that, during ST, Tau was hyperphosphorylated throughout the brain, both at N30 and N. Synthetic torpor leads to a degree of hypothermia (Cerri et al., 2013) which cannot be safely reached by a non-hibernating mammal. Therefore, our results suggest that an evolutionarily ancestral and highly conserved process is active in rat neurons.

Deep hypothermia acts as a main trigger for PP-Tau accumulation, as already described in hibernators (Arendt et al., 2003, 2015) and in mice (Planel et al., 2004, 2007). The accumulation of PP-Tau in hypothermic conditions may depend on the physicochemical characteristics of the main enzymes involved in the phosphoregulation of Tau protein: the glycogen-synthase kinase-3-β (GSK3-β) and the protein-phosphatase-2A (PP2A) (Planel et al., 2004; cf. Gerson et al., 2014). In fact, at low Ta the deactivation of PP2A is faster and larger than that of GSK3-β and, consequently, kinase activity may for some time exceed that of phosphatase (Planel et al., 2004; Su et al., 2008). Specific regulatory mechanisms may also be active in this process, since in hypothermic anesthesia PP-Tau accumulation was shown to be concomitant with an inhibition of the activity of PP2A, while no changes emerged for the activity of several kinases, among which GSK3-β was shown, however, to undergo an inhibitory phosphorylation (Planel et al., 2007). Furthermore, the study of hibernation showed active inhibitory processes on PP2A and GSK3-β during hypothermic bouts (Su et al., 2008; Stieler et al., 2011).

Even though the majority of neural structures analyzed here showed a PP-Tau accumulation peak in the N condition, some specific structures, among whom, the PV and parietal and cerebellar cortices did not, maintaining low levels of AT8 immunoreactivity (AT8-IR). Regional specific differences in AT8-IR have already been described, highlighting a lower AT8-IR in the aforementioned neural structures both in AD patients (Braak et al., 2006; Hu et al., 2017; cf. Furman et al., 2017) and hibernating mammals (Stieler et al., 2011). The functional interpretation of this regional differentiation is rather difficult, and other experiments need to be conducted on this account. However, one suggestion comes from data by Hu et al. (2017), which showed that, in the rat, GSK3-β and PP2A expressions were significantly lower in the cerebellum compared to the hippocampus and cerebral cortex.

Furthermore, in accordance with previous observations (Korneyev et al., 1995; Yanagisawa et al., 1999; Planel et al., 2007), in the present work the Tau-1 staining (that evidences only the non-phosphorylated Tau) was higher in euthermic controls than in ST in all the nervous structures examined.

In conclusion, the strong parallelism of overall PP-Tau accumulation between ST, natural hibernation, and AD, corroborate the possibility of a common mechanism underlying the cellular processes observed in all these cases.

### Resolution of the Accumulation of Phosphorylated Tau Protein During Recovery From ST

Results from the recovery period that follows ST appear of particular interest. Up to now, the reversibility of a strong and diffuse PP-Tau was observed only in hibernating mammals (Planel et al., 2004, 2007; Stieler et al., 2011; Arendt et al., 2015). Our results show that soon after 6 h from the recovery of normothermia following the ST, all the brain structures analyzed solved the AT8-IR, reaching levels that were not different from C.

The dephosphorylation of Tau does not evidently follow the temperature-dependent regulation of the enzymatic activity. In fact, following the formerly described kinetics in the opposite direction, during body rewarming GSK3-β should be the first enzyme to recover its activity (Stieler et al., 2011). Therefore, while returning to normal Tb after the ST bout, there should be an additional period of time during which GSK3-β could act on Tau protein without being contrasted by PP2A (Planel et al., 2004), leading to further phosphorylation. This suggests that the resolution of the PP-Tau condition is an actively regulated process, although the underlying mechanisms are still unknown.

Apart from the involvement of the activity regulation of PP2A (Planel et al., 2007), other mechanisms have been described that could explain the PP-Tau resolution. In fact, PP-Tau may be released by neurons with an activity-dependent process (Serrano-Pozo et al., 2011; Yamada et al., 2011; Pooler et al., 2013), and dephosphorylated in the brain interstitial fluid, which is rich in non-specific phosphatases (Díaz-Hernández et al., 2010). Indeed, it has been observed that patients suffering from AD show much higher levels of Tau than of PP-Tau in CSF (Tapiola et al., 2009). The association between the secretion of PP-Tau and this phosphatasic activity may be an important regulated process that could explain the rapid resolution of PP-Tau following the ST.

In addition to AT8-IR data, Tau-1 immunoreactivity (Tau-1-IR) shows that the dephosphorylation of Tau protein is not complete and the condition reached 38 h after the recovery of normothermia from ST is not exactly the same as that observed in controls, even though rats showed an apparently normal behavior and a normal wake-sleep pattern (Cerri et al., 2013). Notably, a similar dissociation between AT8-IR and Tau1-IR was reported in hibernators (Arendt et al., 2003; Planel et al., 2007). Differently, a normalization of Tau1-IR was observed in rats exposed to cold-water stress by Korneyev et al. (1995) in the aforementioned study.

An interpretation of these findings may lie in the specificity of the antibodies used: Tau-1 recognizes Tau protein only when it is not phosphorylated between residues 189 and 207 (Szendrei et al., 1993; Billingsley and Kincaid, 1997), while AT8 recognizes Tau phosphorylated at Ser202/Thr205/Ser208 (Malia et al., 2016). Our results showed this specular recognition by the two antibodies only in the C and N conditions: when AT8-IR is high, Tau-1-IR is low, and vice-versa. However, in R38 both antibodies showed a low immunoreactivity. This lack of IR of both AT8 and Tau-1 in R38 may be interpreted as a partial dephosphorylation targeted to AT8-specific epitopes of Tau monomers. Notably, if this partial dephosphorylation of Tau is targeted to Ser202, the outcome from ST might even have a neuroprotective effect, since Tau phosphorylation at Thr205 appears to inhibit amyloid-β toxicity in an Alzheimer’s mouse model (Ittner et al., 2016). However, a long-term aversive outcome for neurons may not be excluded from the present data, since 38 h of observation post-ST are not comparable with the long period needed to develop neurodegeneration in animal models of tauopathies (Mustroph et al., 2012; Zhang et al., 2018). Notwithstanding this limit, the analysis of the wake-sleep behavior of rats during the first recovery day following ST (Cerri et al., 2013) suggests that ST does not produce a functional aversive effect.

A further element that may help in understanding the process of resolution of the accumulation of PP-Tau during the recovery from ST comes from a preliminary observation on the possible involvement of microglia in this process, which has been carried out in the present study. It is known that microgliosis, a crucial step in neuroinflammation that is characterized by an activation and proliferation of microglia, is an important hallmark of tauopathies (Ransohoff, 2016; Nilson et al., 2017).

Considering the limitation of our analysis, carried out in only one subject per condition, no relevant modifications in cell morphology, which are considered to be a sign of microglia activation (Stence et al., 2001; Morrison and Filosa, 2013), were observed throughout the experimental protocol in any structure analyzed, as shown in Supplementary Figure S1 for CA3. Although some changes in hippocampal microglia morphology were observed in hibernating hamsters (Cogut et al., 2018; León-Espinosa et al., 2018), a normalization to the euthermic control values occurred at the eighth hour from the end of the torpor bout (Cogut et al., 2018).

However, in the present study we observed a visibly higher microglia density in R6 for all the structures analyzed (Supplementary Figure S2, for CA3), while no such relevant differences were highlighted in R38 compared to C. This suggests that in R6 a transient and mild inflammatory process, apparently reverting toward normal conditions, is occurring. Interestingly, a transient increase of the synthesis of pro-inflammatory cytokines (IL-6, IL-1β) was observed in hibernating hamsters during the 8-h period which followed the torpor bout (Cogut et al., 2018). Microglia are known to be stimulated by extracellular Tau protein (Asai et al., 2015) and in tauopathies they seem to have a role in spreading Tau-tangles throughout the different brain regions (Asai et al., 2015; Bussian et al., 2018). Accordingly, in the R6 condition, when the resolution of AT8-IR is at its maximal rate, a certain level of microgliosis may sustain the clearance of the PP-Tau, that is possibly secreted by neurons (Serrano-Pozo et al., 2011; Asai et al., 2015).

## Conclusion

In the present paper, the induction and reversibility of a widespread PP-Tau accumulation has been described for the first time in a non-hibernating mammal, through an experimental model that makes it possible to take a non-hibernator to a degree of reversible hypothermia comparable to that occurring in hibernators. While the induction of PP-Tau accumulation seems to be due to physicochemical mechanisms, its resolution appears to be actively regulated, possibly involving microglia. Therefore, the physiological mechanism involved in this process can sustain an adaptive neuronal response to extreme conditions, which may however lead to neurodegeneration when particular intensities and durations are exceeded.

## Data Availability

The datasets generated for this study are available on request to the corresponding author.

## Ethics Statement

All the experiments were conducted following the approval by the National Health Authority (decree: No. 112/2018-PR), in accordance with the DL 26/2014 and the European Union Directive 2010/63/EU, and under the supervision of the Central Veterinary Service of the University of Bologna.

## Author Contributions

ML, GZ, RA, and MC contributed to the conception and design of the study. TH, AD, FS, AS, AO, and PC performed the experiments and collected the data. ML performed the statistical analysis. ML, RA, and GZ wrote the first draft of the manuscript. All authors discussed the results, contributed to the manuscript revision, and read and approved the final version of the manuscript for submission.

## Conflict of Interest Statement

The authors declare that the research was conducted in the absence of any commercial or financial relationships that could be construed as a potential conflict of interest.
