# Supplementary material for: Phosphorylation and Dephosphorylation of Tau Protein During Synthetic Torpor
Source: Front Neuroanat. 2019 Jun 6;13:57. doi: 10.3389/fnana.2019.00057 (PMC6563845; doi:10.3389/fnana.2019.00057)

Figure S1. Representative pictures showing the scoring correspondence to the staining intensity observed (Alexa-594): from “-“ (no staining) to “++++” (maximum staining observed). Pictures represent the medial mammillary nuclei stained for AT8. This method was also used for Tau-1 staining. Calibration bar: 100µm.

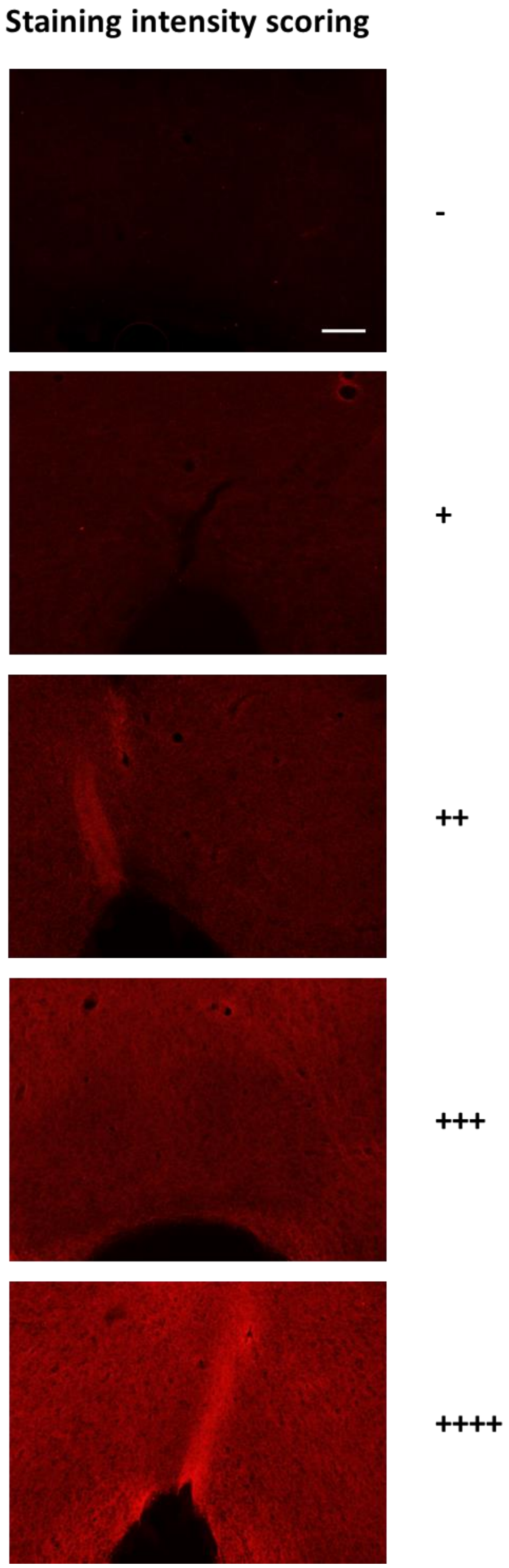

Figure S2. Representative pictures showing microglia, stained with Iba1 (secondary conjugated with Alexa-488). Pictures represent the CA3 field of the hippocampus. C, control (1.9±0.2 cells/100μm^2); N, sample taken at nadir of hypothermia, during the ST (2.5±0.2 cells/100μm^2); R6, sample taken 6h after 35.5 °C brain temperature (Tb) was reached (5.8±0.4 cells/100μm^2); R38, sample taken 38h after reaching 35.5 °C Tb (4.8±0.1 cells/100μm^2). Calibration bar: 50μm.

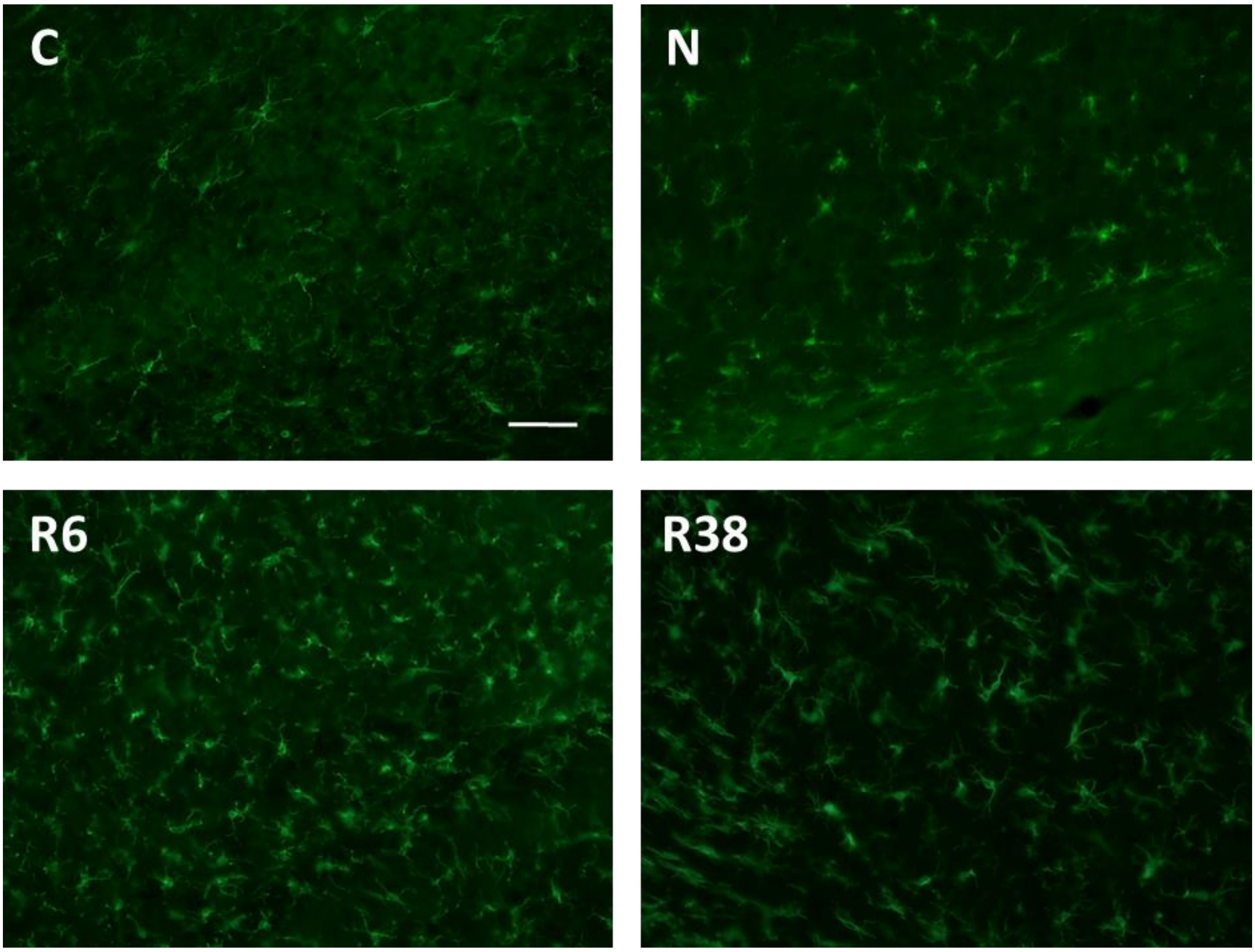

Supplement: Supplementary file 1 [file Data_Sheet_1.pdf]
